# Heart Rate Variability Analysis in Episodic Migraine: A Cross-Sectional Study

**DOI:** 10.3389/fneur.2021.647092

**Published:** 2021-03-23

**Authors:** Lvming Zhang, Shi Qiu, Chunxia Zhao, Peifu Wang, Shengyuan Yu

**Affiliations:** ^1^Department of Neurology, Medical School of Chinese People's Liberation Army, Chinese People's Liberation Army General Hospital, Beijing, China; ^2^Department of Neurology, Aerospace Center Hospital, Beijing, China

**Keywords:** heart rate variability, episodic migraine, cross-sectional study, autonomic nervous function, ictal period, interictal period

## Abstract

**Objective:** It has been reported that autonomic nervous dysfunction is more prevalent in migraineurs. Heart rate variability (HRV) is a commonly used method to evaluate the cardiac autonomic nervous function modulation. However, HRV changes in migraine are still contradictory. The main objective of this study was to explore the potential HRV change patterns in episodic migraine (EM) and whether there were differences in HRV between EM ictal period and the interictal period.

**Patients and Methods:** We conducted a cross-sectional study including 18 patients with EM and 18 age- and sex-matched controls. The characteristics of demographics, some lifestyle factors, and psychological conditions were assessed at baseline. HRVs including time-domain analysis and frequency-domain analysis were performed in all participants. HRV analyses in migraine were recorded not only in the interictal period but also in the ictal period.

**Results:** All the HRV parameters showed a decreased trend in migraine than controls. Time-domain parameters standard deviation of all NN intervals in 24 h (SDNN) and triangular index were significantly lower in the migraine ictal period than controls separately (SDNN, 56.94 ± 22.09 ± 7.76 vs. 135.78 ± 35.16, *p* < 0.001; triangular index, 12.61 ± 3.20 vs. 22.11 ± 6.90, *p* < 0.001). Frequency-domain parameter low-frequency power was also lower in the migraine ictal period than controls (351.28 ± 206.71 vs. 559.61 ± 281.24, *p* = 0.02). SDNN was much lower in the migraine ictal period than migraine interictal period (56.94 ± 22.09 vs. 115.94 ± 46.88, *p* < 0.001). HRV changes during migraine interictal period did not differ from the control group. The correlation analysis revealed a negative correlation between visual analog scale and HRV parameters in the migraine ictal period (*p* = 0.04).

**Conclusions:** The present cross-sectional study indicates that HRV was significantly decreased in EM population especially during the migraine ictal period, which means unbalance of autonomic system in EM. Perhaps larger prospective cohort studies are wanted to validate these findings.

## Introduction

Migraine is a common, disabling neurological disorder that affects ~9.3% of domestic populations in China and ranked as the second in the disability ranking worldwide disorder, leading to substantial personal suffering and impaired quality of life with a significant socioeconomic impact (1, 2). Migraine is characterized by attacks of moderate to severe headache lasting from 4 to 72 h, often unilateral and pulsating, and associated with nausea, vomiting, photophobia, and phonophobia (3). Autonomic symptoms are quite common in migraine and may take many forms. Vomiting and nausea are typical vegetative symptoms, whereas lacrimation, rhinorrhea, and eyelid edema have also been reported in migraine, affecting 27–73% of patients by different estimates (4). Importantly, studies reveal that migraine is associated with increased risk of major cardiovascular disease (CVD) (5–7). In view of this phenomenon, much attention was paid previously to evaluate the autonomic nervous system (ANS) changes (8–13). It has been suggested that autonomic regulation of circulation changed in migraine pain particularly (14–17).

Heart rate variability (HRV), as a non-invasively and effective method in cardiac autonomic tone, has gradually become commonly used to evaluate the autonomic system modulation of cardiac function (4, 18, 19). HRV is a quantitative assessment of variation in heartbeat intervals. Parasympathetic effects of the heart rate are fast, whereas sympathetic modulation are slower (20). Decreased HRV in the time domain has been reported to have a correlation with autonomic impairment and increased mortality and morbidity in various disease states. Exploring the role of HRV in migraine might provide a deeper understanding of the increased risk for CVD in migraineurs.

Although the association between HRV change and migraine has been reported in some literatures, current data on cardiovascular autonomic regulation in migraine are still contradictory. A study reported the root mean square of successive R-R differences (RMSSD) to be higher in migraine patients (21), and another found RMSSD to be lower in migraine (13), whereas others revealed no relationship between HRV and migraine (22–24). Previous studies have emphasized a role for sympathetic hyperactivity with a lower, vaguely mediated HRV in migraine disorders (13, 21). Several studies on high-frequency power (HF) HRV reported no significant differences between migraine patients and healthy controls (22, 25, 26). Most importantly, there is no article describing the HRV changes in migraine in Chinese population as far as we know.

Therefore, we conducted a matched cross-sectional study to assess the relationships between HRV and migraine.

## Patients and Methods

### Study Design and Population

**Figure 1:**
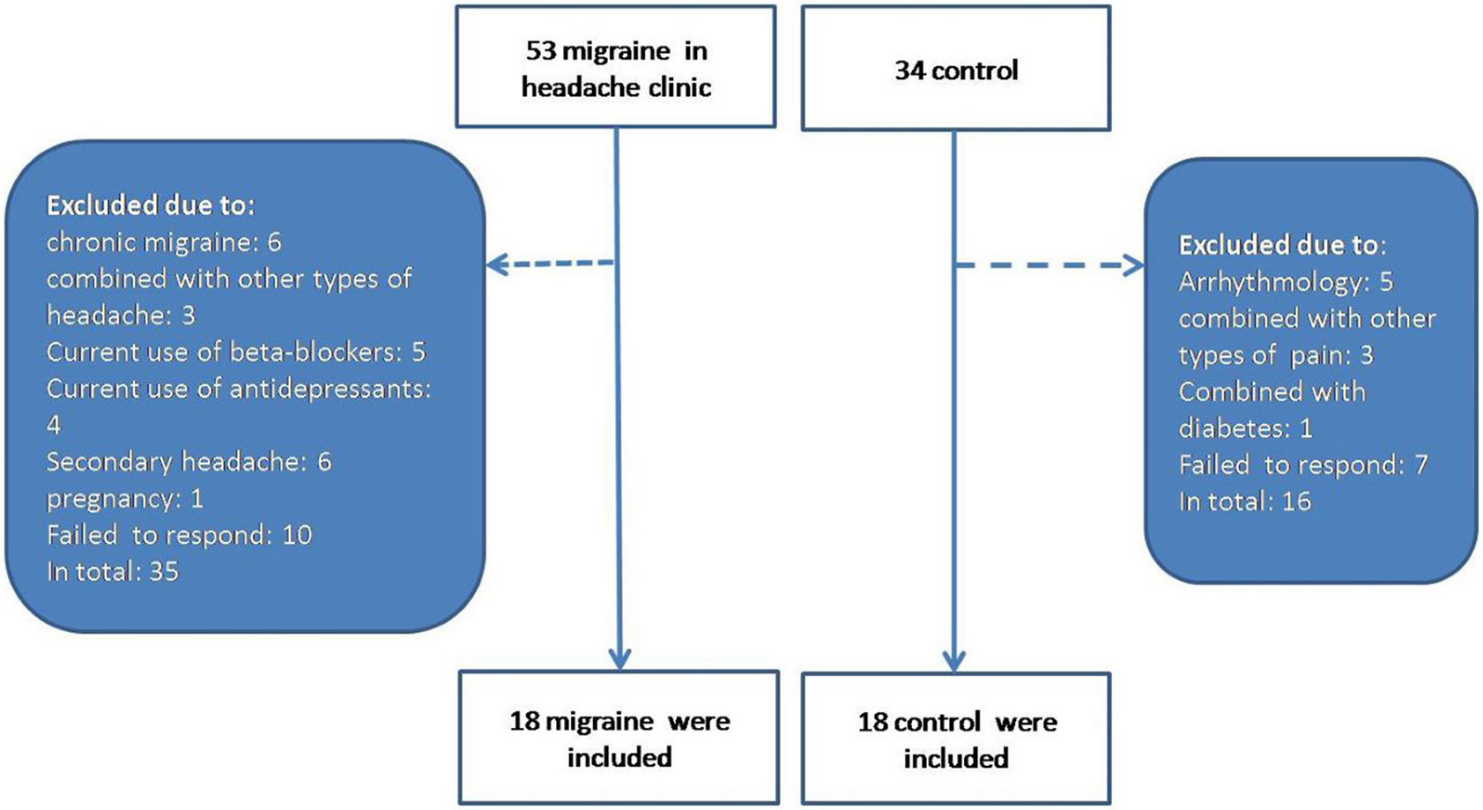
The flowchart of patients' recruitment.

This is a cross-sectional study involving individuals who presented to the Aerospace Center Hospital and underwent dynamic electrocardiogram (ECG) between October 2019 and February 2021. Consecutive patients who were diagnosed with episodic migraine (EM) without aura between 18 and 60 years of age in the Department of Neurology Headache Clinic were enrolled in the case group. A diagnosis of EM without aura was made by experienced doctors in the headache clinic according to the *International Classification of Headache Disorders, Third Edition* (ICHD-3) (3). Individuals with chronic migraine, combined with other types of headache were excluded. Patients with migraine caused by trauma, infection, ischemic stroke, or aortic dissection were excluded from the EM group. These EM patients were matched 1:1 by sex and age with controls without migraine in the health management center and were recruited. The exclusion criteria for all participants were the history of diabetes mellitus; arrhythmia; coronary artery disease or current use of antidepressants anxiolytics, β-blockers, or any type of analgesic medication; self-reported psychiatric conditions; and in lactation or pregnancy. The study was approved by the ethics committee of the Aerospace Center Hospital. We obtained a written informed consent from all subjects, and all procedures in this study adhered to the principles of the Declaration of Helsinki. Finally, 36 participants met the criteria (18 in the case group and 18 in the control group) and were recruited in our study (Figure 1).

### Data Collection

#### Clinical Assessment

All participants received a medical history consultation and standard physical examination. A uniform case report form was used to record the subjects' demographic characteristics and clinical medical history, including age, gender, weight, height, body mass index (BMI), hypertension history, and smoking history. The subjects filled out the questionnaire according to the guidance provided by trained doctors. Hypertension was defined as having a systolic blood pressure (BP) of ≥140 mmHg or diastolic BP of ≥90 mmHg or receiving antihypertensive treatment. Weight and height were used to calculate BMI (weight [kg]/height^2^ [m]). BP was measured using an electronic device. Smoking was assessed by the question “Do you smoke?” Those who answered “yes” were considered as smokers. Perceived pain intensity in EM recording the mean of the pain intensity during the HRV examination was measured with a visual analog scale (VAS) ranging from 0 to 10. The frequency of migraine attacks was measured by the average number of attacks per month in the past 3 months. The duration of migraine was measured in hours, and the average duration of a single migraine episode in the past month was collected.

#### Psychological Evaluation

The psychological evaluation aimed at assessing anxiety and depression. The questionnaire to assess anxiety consisted of Beck Anxiety Inventory (BAI), which is a 21-item scale with a scoring range from 21 to 84 points, with high scores indicating more severe anxiety. In order to measure the severity of depression, we used Beck Depression Inventory (BDI), which is composed of 13 items rated on a four-point scale, and a total score results after ratings summation for the individual items. Higher scores indicate greater depressive symptoms.

#### HRV Measurement

In order to minimize the influence of personal factors on HRV, we selected the same population for HRV measurement during and between migraine episodes. HRVs in migraine and controls were addressed by Holter dynamic ECG in a relatively quiet environment. All participants were instructed not to drink alcohol the evening before the examination; not to exercise, smoke, or consume caffeine 4 h before the experiment; and not to eat 2 h prior to the experiment. In migraineurs, the HRV was collected during both the migraine interictal period and the migraine ictal period. When the migraine attacked, HRVs were recorded in the first 6 h during the migraine attack at least 30 min by using the dynamic ECG. During the interictal period of EM, the HRV was collected at least 24 h after the previous attack and 48 h before the next attack. Participants were asked to remain seated in a relaxed upright position while thinking freely with normal breath rate, remain silent, and not make any sudden movements. After completing questionnaires inquiring about the occurrence of migraine headaches during the previous 3 months, participants were fitted with an ECG recorder (model BI 9800; Boying Medical Instrument Technology, Co., Ltd, Shenzhen, China) worn for 24 h. Six electrodes were placed on the fourth right and left intercostals adjoining the sternum and the left lower ribs. Recordings were analyzed in the cardiovascular function room by two specialist electrocardiographers using an ambulatory ECG system (Boying Medical Instrument Technology, Co., Ltd, Shenzhen, China). The ECG recording was visually inspected and processed offline by using the ECG processing software, which was also used to derive HRV variables. Interference and premature beats in the ECG should be excluded before all tests. The time-domain and frequency-domain analyses including the standard deviation of all NN intervals (SDNN), RMSSD, the percentage of adjacent R-R intervals that differ by more than 50 ms (pNN50), triangular index, HF, low-frequency power (LF), and very low-frequency power (VLF) were adopted to assess the HRV of patients.

### Statistical Analysis

All data were performed by using the software SPSS (version 17.0). There were no missing data on demographic characteristics, history of chronic diseases, and HRV results. In order to compare the distributions of demographic and clinical characteristics between the migraine and control groups, χ^2^ test was used to analyze the categorical variables, which are summarized as frequency and percentage, and the Mann–Whitney test was used to compare the continuous variables, which are presented as means and standard deviation. Pearson correlation was applied for comparative analysis of HRV analysis and clinical characteristics in migraine with the significance level *p* < 0.05. A two-tailed *p* < 0.05 was considered statistically significant.

## Results

### The Baseline Characteristics of Participants

Eighteen patients with EM without aura (female: 13, male: 5; 43.67 ± 17.07 years old) and 18 age- and sex-matched controls (female: 9, male: 9; 42.83 ± 14.96 years old) were finally recruited (Figure 1). The baseline characteristics of participants are summarized in Table 1. Generally, the demographic characteristics including age, gender, BMI, smoking habits, and history of hypertension were not significantly different between the patients with migraine and individuals in the control group. Migraine attack frequency and duration were recorded in all patients in the EM without aura group. Psychological evaluation BAI and BDI showed no statistically significant differences between the two groups.

**Table 1:** Baseline characteristics of the study participants.

**Characteristics**	**Control (*n* = 18)**	**Migraine (*n* = 18)**	***P-value***
**Demographic**			
Age (years), mean ± SD	42.83 ± 14.96	43.67 ± 17.07	0.82
Female, no. (%)	9 (50.00%)	13 (72.22%)	0.15
BMI (kg/m2), mean ± SD	23.43 ± 1.47	23.52 ± 1.91	0.16
Current smoker, no. (%)	6 (33.33%)	4 (22.22%)	0.36
**Clinical characteristics**			
Hypertension, no. (%)	4 (22.20%)	5 (27.78%)	0.50
Systolic blood pressure (mmHg), mean± SD	120.11 ± 11.62	123.89 ± 9.58	0.26
Diastolic blood pressure (mmHg), mean ± SD	68.17 ± 6.17	66.06 ± 5.93	0.71
VAS, mean ± SD	NA	4.89 ± 1.91	
Frequency, per month	NA	4.11 ± 1.84	
Duration, h	NA	22.67 ± 13.20	
BAI, mean ± SD	22.94 ± 1.59	24.11 ± 1.53	0.84
BDI, mean ± SD	2.17 ± 3.78	2.56 ± 2.12	0.22

P-values were calculated by comparing characteristics between the migraine and control.

*BMI, body mass index; VAS, visual analog scale; BAI, Beck Anxiety Inventory; BDI, Beck Depression Inventory; SD, standard deviation*.

### Comparison of the HRV in Patients With Migraine and in the Control Group

All of the HRV parameters, including the time-domain analysis and frequency-domain analysis, have a downward trend in migraine. HRV time-domain analysis revealed significantly lower SDNN and triangular index in the migraine ictal period than in the controls (SDNN, 56.94 ± 22.09 ± 7.76 vs. 135.78 ± 35.16, *p* < 0.001; triangular index, 12.61 ± 3.20 vs. 22.11 ± 6.90, *p* < 0.001). HRV frequency-domain parameter low-frequency power (LF) also demonstrated to be significantly lower in the migraine ictal period than controls (351.28 ± 206.71 vs. 559.61 ± 81.24, *p* = 0.02). We also found the other decreased HRV frequency-domain variability such as RMSSD (*p* = 1.00) and pNN50 (*p* = 0.91) in the EM group, although there is no significant difference from the control group. HF (*p* = 0.06) and VLF (*p* = 0.16) had no differences between the migraine ictal period and controls (Table 2).

**Table 2:** Parameters of HRV in patients with migraine and in control group.

**HRV**	**Control**	**Migraine (*****n*** **=** **18)**		***P*1-values**	***P*2-values**
	**(*n* = 18)**	**Interictal period**	**Ictal period**		
**Time-domain analysis**					
SDNN (ms), mean ± SD	135.78 ± 35.16	115.94 ± 46.88	56.94 ± 22.09	<0.001	<0.001
RMSSD (ms), mean ± SD	31.44 ± 8.79	31.22 ± 10.09	31.44 ± 8.80	1	0.94
pNN50 (%), mean ± SD	10.72 ± 8.63	11.39 ± 8.44	10.39 ± 9.77	0.91	0.74
Triangular index, mean ± SD	22.11 ± 6.90	16.44 ± 10.29	12.61 ± 3.20	<0.001	0.14
**Frequency-domain analysis**					
HF (ms^2^), mean ± SD	294.42 ± 202.04	216.82 ± 137.63	184.26 ± 134.45	0.06	0.48
LF (ms^2^), mean ± SD	559.61 ± 281.24	398.10 ± 197.56	351.28 ± 206.71	0.02	0.49
VLF (ms^2^), mean ± SD	1, 111.02 ± 210.49	1, 073.79 ± 429.44	904.27 ± 569.23	0.16	0.32

*P1 values were calculated by comparing HRV between control group and migraine ictal period; P2 values were calculated by comparing HRV between migraine ictal period and migraine interictal period. Abbreviations: SDNN, standard deviation of all NN intervals in 24 h; RMSSD, the root mean square of successive R-R differences; pNN50, the percentage of adjacent R-R intervals that differ by more than 50 ms; HF, high-frequency power; LF, low-frequency power; VLF, very low-frequency power; SD, standard deviation*.

HRV analysis in the migraine interictal period also showed a decreased trend. However, there was no significant difference between the migraine interictal period and control.

### Comparison of the HRV During and Between Migraine Episodes

Compared with the migraine interictal period, decreased HRV parameters were more prominent during the migraine ictal period. SDNN reveals to be significantly lower (56.94 ± 22.09 vs. 115.94 ± 46.88, *p* < 0.001) in the migraine ictal period than in the migraine interictal period, whereas there were no statistically significant differences in the RMSSD (*p* = 0.092), pNN50 (*p* = 0.76), triangular index (*p* = 0.35), HF (*p* = 0.34), LF (*p* = 0.80), and VLF (*p* = 0.31) between the two groups (Table 2).

### Comparison of the HRV in Different Phases in All the Participants

**Figure 2:**
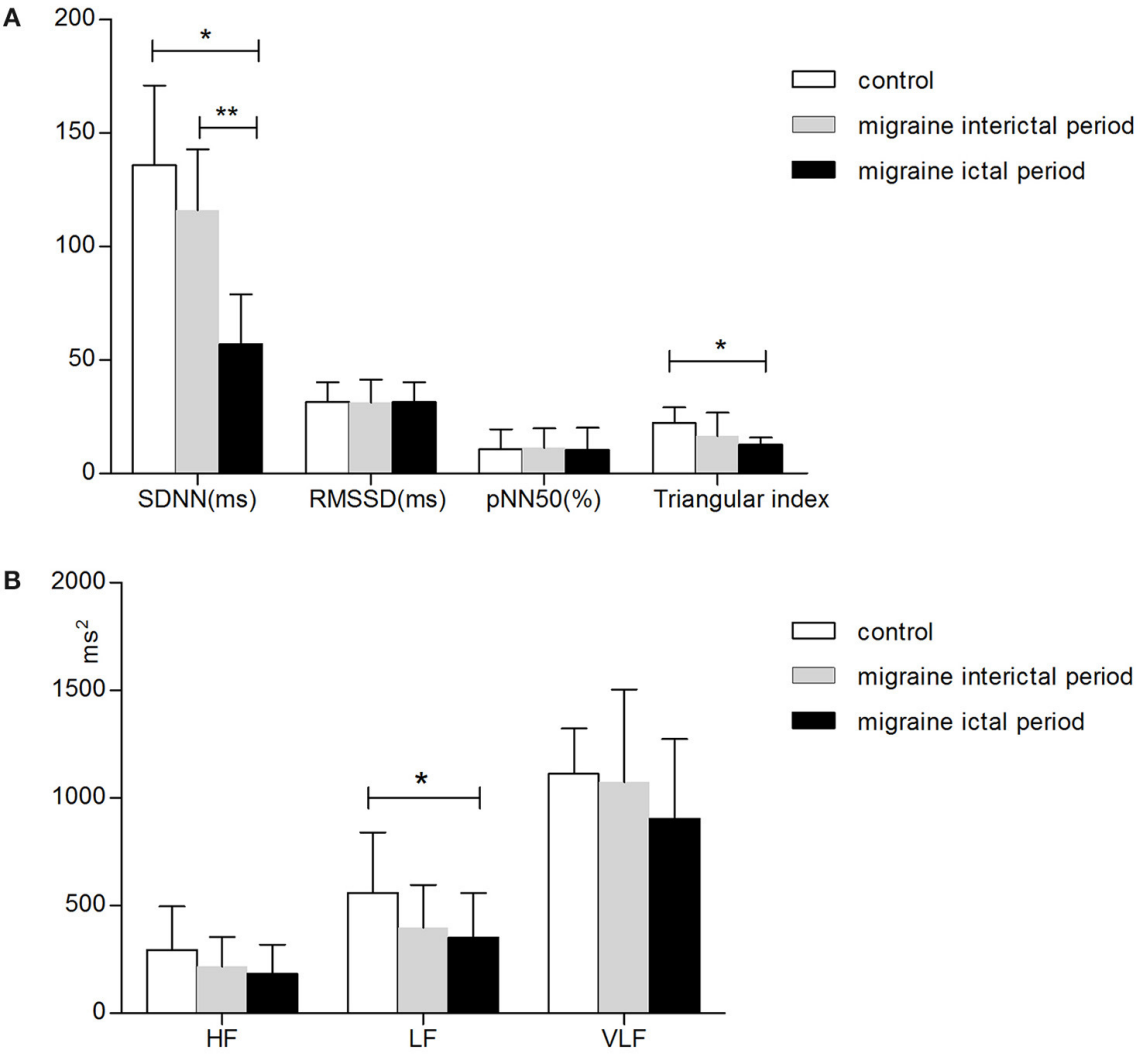
Parameters of HRV in all participants. Panel **(A)** shows the time-domain parameters, and panel **(B)** shows the frequency-domain parameters. HRV parameters were assessed as mean values, whereas the error bars represent the standard error of the mean. *Difference between the migraine ictal period and controls, *p* < 0.05. **Difference between the migraine interictal period and ictal period, *p* < 0.05. Abbreviations: SDNN, standard deviation of all NN intervals in 24 h; RMSSD, the root mean square of successive R-R differences; pNN50, the percentage of adjacent R-R intervals that differ by more than 50 ms; HF, high-frequency power; LF, low-frequency power; VLF, very low-frequency power.

Figure 2 was drawn to better describe the differences in HRV between migraine ictal periods, interictal periods, and control groups. All the denoted parameters in the migraine ictal period differ from the interictal period or controls. The strongest effect (*p* < 0.001) was observed in SDNN when HRVs were analyzed in different phases in migraine patients and controls. Likewise, the triangular index showed significant changes (*p* < 0.001) in the migraine ictal period and the control groups. LF revealed a decreased trend in the migraine ictal periods than control (*p* = 0.02). We also found the other decreased HRV changes in the EM group such as RMSSD, pNN50, HF, and VLF, although there were no significant differences from the control group (Figure 2).

### Correlations Between the Clinical Characteristics and Parameters of HRV

Pearson correlation of different parameters of HRV was carried out to explore the consistency between results obtained in different subjects. The relationships between clinical variables of migraine and HRV were also analyzed. Our correlation analysis revealed that there was a negative correlation between VAS and HRV parameters, which was predominant during the attack of migraine. In the migraine ictal period group, the results of the time-domain analysis strongly correlated with the frequency-domain analysis. Similar strong correlation was also found in the migraine interictal period group, indicating a persistence of these enhanced functions (Table 3).

**Table 3:** Pearson correlation coefficients of HRV in the migraine ictal period (upper part) and the migraine interictal period (lower part).

	**Frequency**	**Duration**	**VAS**	**SDNN**	**rMSSD**	**pNN50**	**Triangular index**	**HF**	**LF**	**VLF**
**Migraine ictal period**										
Frequency	1	**0.49**	0.27	−0.41	−0.05	−0.04	−0.39	−0.04	−0.40	−0.41
		***p*** **=** **0.04**	*p* = 0.28	*p* = 0.09	*p* = 0.83	*p* = 0.87	*p* = 0.11	*p* = 0.86	*p* = 0.10	*p* = 0.09
Duration	**0.49**	1	0.05	−0.03	0.01	−0.14	−0.42	0.08	−0.30	−0.19
	***p*** **=** **0.04**		*p* = 0.83	*p* = 0.92	*p* = 0.96	*p* = 0.57	P=0.08	*p* = 0.76	*p* = 0.23	*p* = 0.44
VAS	0.27	0.05	1	**−0.48**	−0.16	−0.08	**−0.48**	−0.25	−0.20	**−0.50**
	*p* = 0.28	*p* = 0.84		***p*** **=** **0.04**	*p* = 0.53	*p* = 0.74	***p*** **=** **0.04**	*p* = 0.32	*p* = 0.44	***p*** **=** **0.04**
SDNN	–0.41	−0.03	**−0.48**	1	**0.61**	**0.54**	**0.72**	**0.76**	**0.72**	**0.84**
	*p* = 0.09	*p* = 0.92	***p*** **=** **0.04**		***p*** **=** **0.01**	***p*** **=** **0.02**	***p*** **<** **0.001**	***p*** **<** **0.001**	***p*** **<** **0.001**	***p*** **<** **0.001**
rMSSD	−0.05	0.01	−0.16	**0.61**	1	**0.92**	0.35	**0.93**	**0.72**	**0.31**
	*p* = 0.83	*p* = 0.96	*p* = 0.53	***p*** **=** **0.01**		***p*** **<** **0.001**	*p* = 0.15	***p*** **<** **0.001**	***p*** **<** **0.001**	***p*** **=** **0.22**
pNN50	−0.04	−0.14	−0.08	**0.54**	**0.92**	1	**0.50**	**0.89**	**0.66**	0.37
	*p* = 0.87	*p* = 0.57	*p* = 0.74	***p*** **=** **0.02**	***p*** **<** 0.001		***p*** **=** **0.03**	***p*** **<** **0.001**	***p*** **<** **0.001**	*p* = 0.13
Triangular index	−0.39	**−0.42**	**−0.48**	**0.72**	0.35	**0.50**	1	**0.51**	0.43	**0.90**
	*p* = 0.11	***p*** **=** **0.08**	***p*** **=** **0.04**	***p*** **=** **0.00**	*p* = 0.15	***p*** **=** **0.03**		***p*** **=** **0.03**	*p* = 0.08	***p*** **=** **0.00**
HF	−0.04	0.08	−0.25	**0.76**	**0.93**	**0.89**	**0.51**	1	**0.68**	**0.51**
	*p* = 0.86	*p* = 0.76	*p* = 0.32	***p*** **<** **0.001**	***p*** **<** **0.001**	***p*** **<** **0.001**	***p*** **=** **0.03**		***p*** **<** **0.001**	***p*** **=** **0.03**
LF	−0.40	−0.30	−0.20	**0.72**	**0.72**	**0.66**	0.43	**0.68**	1	0.38
	*p* = 0.10	*p* = 0.23	*p* = 0.44	***p*** **<** **0.001**	***p*** **<** **0.001**	***p*** **<** **0.001**	*p* = 0.08	***p*** **<** **0.001**		*p* = 0.12
VLF	−0.41	−0.19	**−0.50**	**0.84**	0.31	0.37	**0.90**	**0.51**	0.38	1
	*p* = 0.09	*p* = 0.44	***p*** **=** **0.04**	***p*** **<** **0.001**	*p* = 0.22	*p* = 0.13	***p*** **<** 0.001	***p*** **=** **0.03**	*p* = 0.12	
**Migraine interictal period**										
Frequency	1	0.39	0.19	−0.12	−0.22	−0.06	−0.11	−0.14	−0.20	−0.35
		*p* = 0.11	*p* = 0.46	*p* = 0.63	*p* = 0.38	*p* = 0.80	*p* = 0.65	*p* = 0.57	*p* = 0.42	*p* = 0.15
Duration	0.39	1	−0.10	**0.49**	0.22	0.18	**0.50**	0.24	0.2	0.45
	*p* = 0.11		*p* = 0.70	***p*** **=** **0.04**	*p* = 0.38	*p* = 0.48	***p*** **=** **0.03**	*p* = 0.35	*p* = 0.42	*p* = 0.06
VAS	0.19	−0.10	1	−0.05	0	0.14	0.14	0.1	−0.28	−0.37
	*p* = 0.46	*p* = 0.70		*p* = 0.83	*p* = 0.99	*p* = 0.57	*p* = 0.59	*p* = 0.71	*p* = 0.26	*p* = 0.13
SDNN	−0.12	0.49	−0.05	1	0.26	0.2	**0.78**	0.26	0.24	**0.49**
	*p =* 0.63	*p* = 0.04	*p* = 0.83		*p* = 0.29	*p* = 0.43	***p*** **=** **0.00**	*p* = 0.29	*p* = 0.34	***p*** **=** **0.04**
rMSSD	−0.22	0.22	0	0.26	1	**0.96**	0.27	**0.97**	**0.73**	0.13
	*p* = 0.38	*p* = 0.38	*p* = 0.99	*p* = 0.29		***p*** **<** **0.001**	*p* = 0.29	***p*** **<** **0.001**	***p*** **<** **0.001**	*p* = 0.59
pNN50	−0.06	0.18	0.14	0.2	**0.96**	1	0.26	**0.97**	**0.61**	−0.09
	p = 0.80	*p* = 0.48	*p* = 0.57	*p* = 0.43	***p*** **<** **0.001**		*p* = 0.29	***p*** **<** **0.001**	***p*** **=** **0.01**	*p* = 0.72
Triangular index	−0.11	0.50	0.14	**0.78**	0.27	0.26	1	0.36	0.06	0.22
	*p* = 0.65	*p* = 0.03	*p* = 0.59	***p*** **<** **0.001**	*p* = 0.29	*p* = 0.29		*p* = 0.14	*p* = 0.81	*p* = 0.39
HF	−0.14	0.24	0.1	0.26	**0.97**	**0.97**	0.36	1	**0.71**	0.06
	*p* = 0.57	*p* = 0.35	*p* = 0.71	*p* = 0.29	***p*** **<** **0.001**	***p*** **<** **0.001**	*p* = 0.14		***p*** **<** **0.001**	*p* = 0.80
LF	−0.20	0.2	−0.28	0.24	**0.73**	**0.61**	0.06	**0.71**	**1**	0.51
	*p* = 0.42	*p* = 0.42	*p* = 0.26	*p* = 0.34	***p*** **<** **0.001**	***p*** **=** **0.01**	*p* = 0.81	***p*** **<** **0.001**		***p*** **=** **0.03**
VLF	−0.35	0.45	−0.37	**0.49**	0.13	−0.09	0.22	0.06	**0.51**	1
	*p* = 0.15	*p* = 0.06	*p* = 0.13	***p*** **=** **0.04**	*p* = 0.59	*p* = 0.72	*p* = 0.39	*p* = 0.80	***p*** **=** **0.03**	

*VAS, visual analog scale; SDNN, standard deviation of all NN intervals in 24 h; rMSSD, the root mean square of successive R-R differences; pNN50, the percentage of adjacent R-R intervals that differ by more than 50 ms; HF, high-frequency power; LF, low-frequency power; VLF, very low-frequency power. Significant correlation is highlighted by bold fonts*.

## Discussion

The main finding of this cross-sectional study assessing HRV in migraine is that HRV parameters are significantly lower in EM. Interestingly, this dysfunction is more pronounced in the migraine ictal period but not in the migraine interictal period compared with the matched controls. It looks like that SDNN, triangular index, and LF are more sensitive to respond to this autonomic dysfunction in all the parameters. SDNN and triangular index are thought to reflect the activities of the parasympathetic nervous system. LF power has been reported to be modulated by both sympathetic and parasympathetic activities. Thus, we conclude that the parasympathetic nervous system dysfunction is dominant in EM, while sympathetic nervous system dysfunction also exists. The correlation analysis of the clinical variables of migraine and HRV indicated that HRV parameters decreased further with the increase of VAS in the migraine ictal period. The correlation analysis between time-domain analysis and frequency-domain analysis supports this reliability. Based on our findings, EM has cardiac autonomic system dysfunction in the migraine ictal period. And we hypothesize that effective prevention and treatment of migraine attacks may be able to reduce the cardiac impact in EM.

There are some studies describing the association between migraine and cardiac autonomic function in the past 20 years. Reduced parasympathetic activity with sympathetic predominance has previously been found in migraine patients (22, 23, 27). Most of the studies focused on monitoring the ANS during migraine interictal period. In our study, only EM without aura was brought into study, and no differences were found between the interictal period and controls. A previous study showed reduced SDNN, suggesting sympathetic hyperactivity in migraine with aura during the migraine interictal period, whereas no significant difference was found in migraine without aura (9). An American cohort study evaluated the RR variation, Valsalva maneuver, and cardiovascular reactivity in the migraine ictal period, revealing migraineurs with disabling attacks may be prone to ANS hypofunction (27). We speculate that aura symptoms and a disabling attack may be more directly related to autonomic dysfunction.

At the same time, similar to our results, some studies revealed no difference in migraine. Researchers evaluated the autonomic system function by using HRV and skin conductance responses in EM and controls; no significant differences were found (28). Researchers used vertical tilt test, Valsalva maneuver, grip test, cold-stress vasoconstriction, BP variability, and arterial baroreflex to examine the autonomic regulation in episodic and chronic forms of migraine. The cardiac regulation remains largely unchanged in both the episodic and chronic migraines (11). Studies in Japan also postulate no HRV change in migraine (10). In these studies, HRV should be a relatively stable marker to evaluate the ANS.

A few studies have attempted to explore autonomic nervous function changes during migraine attacks. A Turkey study of 30 patients of migraine revealed particular RR and corrected QT intervals; these abnormalities will be absent or less prominent during pain-free intervals (14), but there are no HRV analysis results. Mustafa et al. found that migraine attacks are associated with an increase in ventricular repolarization parameters compared with attack-free periods possibly because of the dysregulation of the ANS (17). Similar to our study, autonomic system function is damaged in migraine attack. The novelty of our study is the application of relatively objective quantitative HRV analysis to verify this view.

The mechanisms underlying the HRV change patterns in migraine are yet to be fully explored. Lower vague mediated HRV has been reported in chronic pains, including chronic neck pain (29), complex regional pain (30), chronic pelvic pain (31), and fibromyalgia (9, 32, 33). Scholars speculated that the vagus-mediated reduction of HRV in migraine patients might be related to the periaqueductal gray (PAG), which is an important area involved in descending inhibitory modulation of pain (12). In the treatment of chronic pain, stimulation of the ventral PAG region can reduce pain intensity and increase HRV (34). Because of this, vagus nerve stimulation in people with chronic pain (35, 36) including migraine (37) is gradually recognized. Whether stimulation in the ventral PAG increases HRV to reduce migraine-related complications such as CVDs should be confirmed further. It is consistent with our research speculation. In addition, the presence of altered neurotransmitters, such as serotonin and noradrenaline, affects the development of autonomic dysfunction in migraine patients (38).

Interestingly, the HRV changes just occurred during the migraine attack period, but not the interictal period. And we performed Pearson correlation analysis, which revealed HRV parameters decreased with the increase of VAS in the migraine ictal period. Moreover, a previous study reported that migraineurs with no cardiovascular risk factors can also experience coronary artery spasm during a migraine attack (39). Based on this phenomenon, we hypothesized that it is the pain intensity that induced coronary artery spasm during migraine attack. This is consistent with the results of the correlation analysis in our study.

Our study has some highlights. First, this is the first study to report the HRV evaluation in EM in Chinese population. Second, few previous studies have conducted HRV evaluation at the migraine ictal period. We conducted a cross-sectional study not only to evaluate HRV analysis during the migraine ictal period and interictal period, but also compared with a normal control group. Third, our study used an objective quantitative HRV analysis to clarify the presence of cardiac autonomic nervous dysfunction during the attack period of EM without aura. This finding will provide an objective basis for clinical treatment to reduce the incidence of migraine. At the same time, there are some limitations to the present study. First, given the demographic characteristics of the study population, findings are not generalized to EM with aura and patients with coronary heart disease, arrhythmia, and diabetes. Second, this was a single-center study that recruited patients; therefore, we cannot exclude selection bias for patients. Third, there is a low sample in the present study because of difficult evaluation during the migraine ictal period. Thus, the results should be confirmed in a larger study in the future. Finally, although it is relatively well-established that SDNN, triangular index, and LF power are indexes of autonomic system–mediated HRV, the interpretations of SDNN and triangular index in migraine are still less clear. Therefore, further study regarding HRV in EM is still needed in the future.

## Conclusion

The present study illustrates the HRV in EM in Chinese population. HRV analysis shows an imbalance of the autonomic system with a lower SDNN, triangular index, and LF power during the attack of migraine. Considering the prognostic significance of SDNN, triangular index, and LF power, it is necessary to closely monitor migraineurs for adverse cardiovascular outcomes. Much more studies of regulating HRV therapy with the aim to observe disability and complications of migraine are wanted.

## Data Availability Statement

The raw data supporting the conclusions of this article will be made available by the authors, without undue reservation.

## Ethics Statement

Our study was approved by the Ethics Committee of the Aerospace Center Hospital. We obtained a written informed consent from all participants.

## Author Contributions

LZ conceived the study, participated in the design, performed statistical analyses and drafted the manuscript. SQ and CZ collected the data, and drafted the manuscript. LZ and SQ analyzed and interpreted the HRV data results. PW and SY participated in the design and helped to revise the manuscript critically for important intellectual content. All authors read and approved the final manuscript and reviewed this manuscript.

## Conflict of Interest

The authors declare that the research was conducted in the absence of any commercial or financial relationships that could be construed as a potential conflict of interest.

## Abbreviations

BMI: body mass index
VAS: visual analog scale
BAI: Beck Anxiety Inventory
BDI: Beck Depression Inventory
HRV: heart rate variability
ANS: autonomic nervous system
ICHD-III beta: *International Classification of Headache Disorders, Third Edition* (beta version)
SDNN: standard deviation of all NN intervals in 24 h
RMSSD: the root mean square of successive R-R difference
pNN50: the percentage of adjacent R-R intervals that differ by more than 50 ms
HF: high-frequency power
LF: low-frequency power
VLF: very low-frequency power
OR: odds ratio
CI: confidence interval.
